# Correlations Between Structural Brain Abnormalities, Cognition and Electroclinical Characteristics in Patients With Juvenile Myoclonic Epilepsy

**DOI:** 10.3389/fneur.2022.883078

**Published:** 2022-05-16

**Authors:** Jun Zhang, Dan Wu, Haoran Yang, Hongjuan Lu, Yichen Ji, Huixin Liu, Zhenxiang Zang, Jie Lu, Wei Sun

**Affiliations:** ^1^Department of Neurology, Xuanwu Hospital Capital Medical University, Beijing, China; ^2^Department of Radiology and Nuclear Medicine, Xuanwu Hospital, Capital Medical University, Beijing, China

**Keywords:** juvenile myoclonic epilepsy, magnetic resonance imaging, structural abnormalities, cognitive function, electroencephalogram

## Abstract

**Objective:**

To explore the structural brain abnormality and its relationship with neuropsychological disorders and electroclinical characteristics in juvenile myoclonic epilepsy (JME) patients.

**Methods:**

Sixty-seven patients diagnosed with JME and 56 healthy controls were enrolled. All subjects underwent MRI using T1-weighted 3D brain structural images with 1 mm thickness. Voxel-based morphometry (VBM) and surface-based morphometry (SBM) analyses were performed. They also underwent a series of neuropsychological tests to assess cognitive function. The correlation analyses were conducted between structural changes, neuropsychological outcomes, and electroclinical features.

**Results:**

The gray matter concentration (GMC) was decreased in the bilateral pre-central and post-central gyrus, right anterior cingulate gyrus, left posterior orbital region, bilateral occipital regions, bilateral hippocampus and bilateral caudate nucleus in the JME groups (corrected *P* < 0.05). The evaluation of gray matter volume (GMV) showed significant decrease respectively in bilateral pre-central and post-central gyrus, left paracentral lobule, left orbital gyrus, left amygdala, left basal ganglia and left thalamus of JME patients (*P* < 0.05). The cortex thicknesses of the right inferior temporal gyrus, right insular gyrus, and right cingulate gyrus had negative correlations with the disease duration significantly. At the same time, the whole-brain white matter volume was positively associated with the course of the disease (*P* < 0.05). Patients with persistent abnormal EEG discharges had significantly less whole-brain gray matter volume than JME patients with normal EEG (*P* = 0.03). Correlation analyses and linear regression analyses showed that, in addition to the gray matter volumes of frontal and parietal lobe, the temporal lobe, as well as the basal ganglia and thalamus, were also significantly correlated with neuropsychological tests' results (*P* < 0.05).

**Conclusion:**

The JME patients showed subtle structural abnormalities in multiple brain regions that were not only limited to the frontal lobe but also included the thalamus, basal ganglia, parietal lobe, temporal lobe and some occipital cortex, with significant involvement of the primary somatosensory cortex and primary motor cortex. And we significantly demonstrated a correlation between structural abnormalities and cognitive impairment. In addition, the course of disease and abnormal discharges had a specific negative correlation with the structural changes.

## Introduction

Juvenile myoclonic epilepsy (JME) is a specific idiopathic generalized epilepsy (IGE) syndrome with a prevalence of 5–10% of all epilepsies and 18% of IGE (1). It is characterized by an age-specific onset of epilepsy with myoclonic jerks, generalized tonic-clonic seizures, and, less frequently, absences (2). Most patients with JME respond well to antiseizure medication (ASM). A high response rate of 80% was reported in patients treated with sodium valproate (VPA), which is the first-line choice in men with JME (3). On the other hand, there is a common belief that JME requires lifelong treatment because of an extremely high rate of relapse at attempts to terminate medication (2). For quite some time, JME patients' brain were considered “normal” on visual inspection. However, the development of MRI acquisition technology and post-processing methods of brain structures revealed subtle abnormalities in brains of JME patients since the first paper published by Swartz in 1996 (4). The structural and functional changes were mainly located in frontobasal areas (5), the prefrontal cortex, the hippocampus, and the corpus callosum (6), or in bilateral superior mesio-frontal regions as well as the thalami (7), mostly implicating fronto-cortico-thalamic regions and their connections (8–11). Cognitive functions in IGE, especially JME, is of increasing research attention in recent years. Many researches indicated specific profiles of cognitive impairment, particularly encompassing functions reliant on frontal lobe processing (12). Pilsipher and collaborators suggested that the same circuitry accounting for seizure generation in JME may also mediate impairment of executive skills (13). Other studies attempted to identify the neural correlation of cognitive traits in JME by means of structural imaging. Changes in microstructural integrity of the supplementary motor area were associated with reduced performance on an expressive language task, while both gray matter volume and microstructural integrity of the posterior cingulate cortex related to mental flexibility (14). Caeyenberghs reported that connectivity between post-central gyrus and precuneus was positively associated with verbal IQ, expressive language as well as verbal memory scores (15). Compared with the evidence obtained from functional imaging studies, the results provided by previous structural imaging research were short of some consistency.

There have been numerous studies on imaging and cognitive function of JME patients. Based on the mechanism of the fronto-thalamo-cortical network dysfunction, the research mainly focused on the abnormal structure of the frontal lobe and thalamus. The results of various structural and functional MRI studies were diverse, and the abnormal brain regions and specific impaired cognitive domains were still controversial.

Our study aimed to characterize the structural brain abnormalities and cognitive impairments in JME patients and explore the relationship between structure and cognitive deficits by studying a larger sample size of the JME population than other research.

## Materials and Methods

### Participants

Sixty-seven patients with JME were recruited at the outpatient clinics of Xuanwu Hospital Capital Medical University from January 2013 to December 2019. Ethical approval was granted by Xuanwu Hospital Capital Medical University Ethics Committees and written informed consent was obtained from all patients. Inclusion criteria were as follows: (1) unequivocal diagnosis of JME based on criteria defined in the revised Classification of Epilepsies and Epileptic Syndromes (3); (2) no history of neurological or intellectual deficits; and (3) normal clinical MRI. In addition, a total of 56 healthy control subjects without neurological and psychiatric illnesses were recruited.

### MRI Acquisition

MRI scanning was performed with a three Tesla scanner (Magnetom Symphony, Siemens Medical Systems AG, Erlangen, Germany) with a 12-channel standard head coil. The parameters for T1-weighted 3D brain structural images were as follows: SPGR sequence, FOV = 256 × 256 mm^2^, matrix = 256 × 256, slice thickness = 1 mm, gap = 0, slice number = 192, repetition time (TR) = 6.9 ms, echo time (TE) = 2.98 ms, inversion time (TI) = 450 ms, flip angle = 12°, and voxel size = 1 × 1 × 1 mm^3^.

### Image Processing

Image processing was performed on Statistical Parametric Mapping 12 software (SPM12; Wellcome Department of Cognitive Neurology, University College, London, UK) using CAT 12 toolbox (Departments of Psychiatry and Neurology, University of Jena, Thuringia, Germany). The T1 images were spatially registered to the MNI template, and then were segmented into white matter (WM), gray matter (GM) and CSF. Bias correction would be performed to remove intensity non-uniformities. During the normalization process, the modulated images were resampled and preserved at 1.0 mm isotropic resolution. Finally, for each individual, we would obtain a smoothed GM volumetric map by spatial smoothing the normalized GM images with an 8 mm FWHM Gaussian kernel. The total intracranial volume (TIV) of each subject were also calculated and used as a covariate for further statistical analyses. We defined ROIs according to the Brainnetome (BN) atlas (http://atlas.brainnetome.org), which contains 246 brain regions in both hemispheres (16). At the same time of segmentation, the surface and thickness estimation were performed simultaneously. The resample size was 164k mesh while the smoothing filter size in FWHM was 15 mm.

### Neuropsychological Testing

Sixty-seven JME patients and 56 healthy controls underwent a series of neuropsychological tests to evaluate language, memory, attention, calculation, psychological speed and visuospatial functions. These tests were computerized on a web-based system called the “Online Psychological Experiment System (OPES)” (47.95.214.92/lattice/). The tests are as follows: General intelligence (Raven's Standard Progressive Matrices); Attention and executive function (Visual Research Task and Visual Tracing Task); Executive function [Wisconsin Card Sorting Test (WCST)]; Memory [Auditory Verbal Learning Test (AVLT) Immediate Memory, AVLT Delayed Memory, AVLT Recognition Memory, Digit Span Test, Digital n-back Test and Spatial n-back Test]; Psychomotor speed and language processing (Choice Reaction Time and Word Discrimination Test); Visuospatial processing and arithmetic calculation (Three-dimensional Mental Rotation and Complex Subtraction Test); Visual perception (Visual Perception Task).

### Electroencephalogram Recordings and Analysis

Electroencephalogram (EEG) was recorded with 32-channel and a sampling frequency of 1,024 Hz using the standard international 10–20 system (Bio-logic, America). The EEG recordings were analyzed in a referential montage of A1 and A2 earlobe electrodes. All patients underwent at least 4 h of video EEG monitoring to record awake period and sleep EEG. Typical EEG patterns was defined in presence of generalized symmetric discharges of single or polyspike and slow wave (S/PS-SW). As well as lateralized or asymmetric sharp waves, EEG asymmetries included unilateral S/PS-SW discharges, discharges with unilateral onset becoming generalized, or discharges with above 50% voltage asymmetries (confirmed in more than one recording) (17).

### Statistics

SPSS 26.0 (IBM Co., Armonk, NY, USA) was used for statistical processing of the data. Group differences in demographic measures were tested using the independent samples *t*-test and the chi-square analyses for continuous and categorical variables, respectively. To correct for multiple comparisons, the results of GMC were corrected with a false discovery rate (FDR) correction at a height threshold of a *P* < 0.05. Localized regional differences of gray matter volumes and cortical thickness were analyzed using ANCOVA, with age, gender, and TIV as covariates. The analyses of the relationship between the structures' parameters with disease duration, age of onset of seizures were performed using Pearson's correlation coefficients. The relationship between the structural changes with neuropsychological tests' scores was conducted using correlation analysis and linear regression analysis. *P*-value < 0.05 was considered statistically significant.

## Results

### Demographic and Clinical Data

A total of 67 patients (male, *n* = 29) were included in the analysis, with a mean age of 23.07 ± 5.89 years (range 12–37 years) and epilepsy duration of 8.67 ± 5.77 years old (range 1–20 years). Age at seizure onset ranged from 7 to 28 years old, 48 of them had their first seizure attack between 12 to 18 years old, which is the peak age of onset. Most patients had their first seizure type as myoclonic seizures, then followed GTCS, and only a minority of patients presented with absence seizures in the early period of their clinical course. There were 43 (64.2%) JME patients who had both MS and GTCS, which accounted for the vast majority of the JME subjects. Sodium valproate (VPA), levetiracetam (LEV), or lamotrigine (LTG) was administrated as a monotherapy in 44 patients, and 23 patients received a regimen of a combination of two or three drugs. Detailed demographic and clinical characteristics are summarized in Table 1.

**Table 1:** Clinical data of JME patients and healthy controls.

	**JME (*n* = 67)**	**Controls (*n* = 56)**	***P*-value**
**Gender**			
Male, *n* (%)	29(43.3)	20 (35.7)	0.393
Female, *n* (%)	38(56.7)	36(64.3)	
Age, years, mean ± SD, range	23.07 ± 5.89, 12–37	26.77 ± 4.68, 13–36	<0.001
Education, years, mean ± SD, range	15.18 ± 3.50, 7–22	16.16 ± 2.21, 6–22	0.062
Age at seizure onset, years, mean ± SD, range	14.36 ± 3.48, 7–28		
Duration of epilepsy, years, range	8.67 ± 5.77, 1–20		
Seizure remission, months, range	16.49 ± 17.38, 0–108		
Family history of epilepsy, *n* (%)	5 (7.5)		
History of febrile seizures, *n* (%)	6 (9.0)		
**First type of seizures**, ***n*** **(%)**			
MS	35 (52.2)		
GTCS	30 (44.8)		
AS	2 (3.0)		
**Types of seizure**, ***n*** **(%)**			
MS	4 (5.9)		
MS and GTCS	43 (64.2)		
MS, GTCS and AS	20 (29.9)		
**ASM**, ***n*** **(%)**			
VPA	17 (25.4)		
LEV	25 (37.3)		
LTG	2 (3.0)		
Polytherapy	23 (34.3)		

*MS, myoclonic seizure; GTCS, generalized tonic-clonic seizure; AS, absence seizure; ASM, antiseizure medication; LEV, levetiracetam; VPA, sodium valproate; LTG, Lamotrigine*.

### Image Analysis

#### Voxel-Based Morphometry

**Figure 1:**
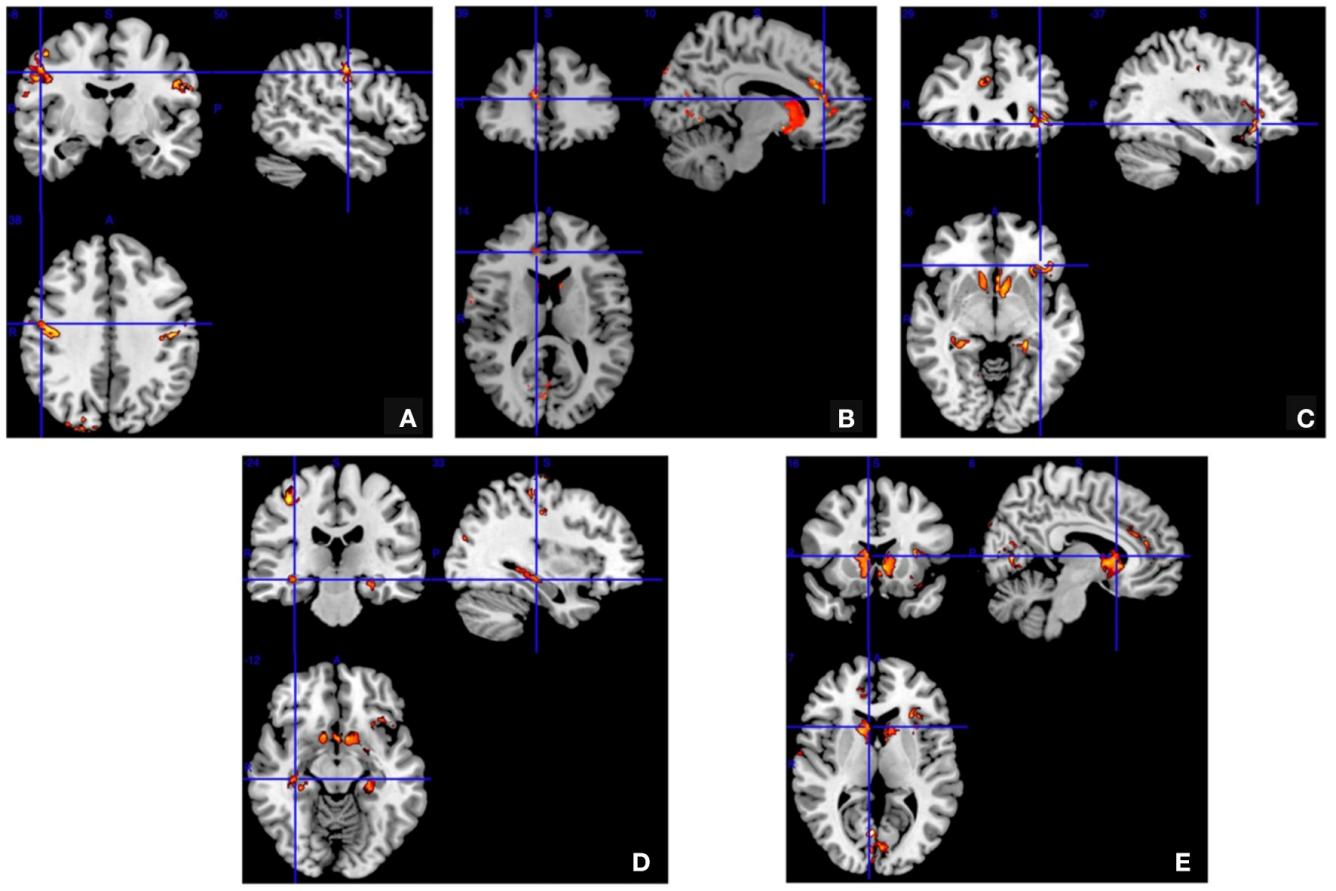
Voxel-based morphometry. The gray matter concentration was decreased in the bilateral pre- and post-central gyrus **(A)** right anterior cingulate gyrus **(B)** left posterior orbital region **(C)** bilateral hippocampus **(D)** and bilateral caudate nucleus and bilateral occipital regions **(E)** in the juvenile myoclonic epilepsy patients compared to the normal controls (false discovery rate corrected *P* < 0.05).

The gray matter concentration was decreased in the bilateral pre- and post-central gyrus (especially in the right hemisphere), the right anterior cingulate gyrus, the left posterior orbital region, the bilateral hippocampus, caudate nucleus and the bilateral occipital regions in the JME group (*P* < 0.05; Figure 1). But no brain regions showed an increased gray matter concentration.

#### The Volumetry Evaluation of Gray Matter

The volumetry evaluation of gray matter was shown in Table 2. When compared to control subjects, JME patients had significant GMV reductions in the bilateral pre- and post-central gyrus, left orbital gyrus, left paracentral lobule, left basal ganglia, left amygdala and left thalami. At the same time, JME patients had significant GMV increases in the left precuneus and right parahippocampal gyrus (*P* < 0.05).

**Table 2:** Volumetry evaluation of gray matter differences between patients and controls.

**Lobe**	**BNA label**	**MNI coordinates**	**Volumes (Mean** **±SD) (cm**^**3**^**)**		***P*-Value^†^**
			**JME**	**Controls**	
**JME patients** **<** **controls**					
Frontal lobe	OrG_L_6_6	−41, 32, −9	2.25 ± 0.31	2.33 ± 0.25	0.007
	PrG_L_6_2	−31; −9; 58	2.58 ± 0.43	2.76 ± 0.44	0.009
	PrG_L_6_4	−13; −20; 73	2.00 ± 0.23	2.14 ± 0.27	0.002
	PCL_L_2_1	−8; −38; 58	1.25 ± 0.16	1.32 ± 0.17	0.013
	PrG_R_6_1	55; −2; 33	2.19 ± 0.27	2.31 ± 0.25	0.031
Parietal lobe	PoG_L_4_1	−50; −16; 43	3.10 ± 0.44	3.24 ± 0.35	0.037
	PoG_R_4_1	50; −14; 44	3.08 ±0.32	3.29 ± 0.34	0.002
Subcortical area	Amyg_L_2_2	−27; −4; −20	0.44 ± 0.05	0.44 ± 0.04	0.048
	BG_L_6_1	−12; 14; 0	1.99 ± 0.25	2.08 ± 0.19	0.048
	Tha_L_8_5	−16; −24; 6	0.946 ± 0.098	0.955 ± 0.075	0.015
	Tha_L_8_7	−12; −22; 13	0.822 ± 0.099	0.824 ± 0.074	0.025
**JME patients** **>** **controls**					
Parietal lobe	PCun_L_4_3	−12; −67; 25	3.70 ±0.56	3.52 ± 0.49	0.006
Temporal lobe	PhG_R_6_4	19; −10; −30	0.73 ± 0.11	0.72 ± 0.08	0.025

*L, left; R, right; OrG, Orbital gyrus; PrG, pre-central gyrus; PCL, Paracentral Lobule; PoG, post-central gyrus; Amyg, Amygdala; BG, Basal Ganglia; Tha, Thalamus; PCun, Precuneus; PhG, Parahippocampal Gyrus; BNA, Brainnetome Atlas. MNI, Montreal Neurologic Institute*.

† *Covariance analysis: TIV (total intracranial volume), age and gender as covariates*.

#### Surface-Based Morphometry

As shown in Supplementary Table 1, compared with normal controls, the cortical thickness involved the widest range of brain when compared with the control group. Thickness decreased in almost the entire frontal region of the brain in JME patients, such as bilateral superior frontal gyrus (SFG), middle frontal gyrus (MFG), inferior frontal gyrus (IFG), orbital gyrus (OrG) and pre-central gyrus (PrG). Paracentral lobules located in the medial surface of the bilateral hemisphere were no exception. And the right temporal lobe was more involved than the left temporal lobe, especially the right fusiform gyrus and right parahippocampal gyrus. In the parietal lobe, we found a thickness decrease in bilateral superior parietal lobules, inferior parietal lobules and posterior central gyrus, as well as in precuneus. Finally, the abnormality of insular lobe, cingulate gyrus and occipital region were more prominent in the right hemisphere.

### The Relationship Between EEG Characteristics and Brain Volumes in JME Patients

EEG features of our JME series were detailed in Table 3. Eighteen patients (26.9%) showed normal EEG findings. Meanwhile, a symmetrical EEG pattern was evident in 37 patients (55.2%) while EEG asymmetries were detected in 12 patients (17.9%) which consisted of asymmetric or, more rarely, lateralized sharp waves and spike/polyspike-slow wave complexes. There was significant difference in N-GMV between patients with normal and abnormal EEG patterns (*P* = 0.03; Table 4).

**Table 3:** EEG findings in JME patients.

**Parameter**	**Number (%)**
Normal	18 (26.9)
Abnormal	49 (73.1)
S/PS-SW^a^	37
EEG asymmetries^b^	12

a *S/PS-SW, spike/polyspike-slow wave discharges*.

b *Including lateralized or asymmetric sharp wave, unilateral S/PS-SW discharges, discharges with unilateral onset that becoming generalized, or discharges with above 50% voltage asymmetries*.

**Table 4:** The relationship between EEG characteristics and brain volumes.

**Volume**	**Normal (18)**	**Abnormal (49)**	***P*-Value**	**Symmetrical (37)**	**Asymmetrical (12)**	***P*-Value**
	**Mean** **±SD**	**Mean** **±SD**		**Mean** **±SD**	**Mean** **±SD**	
N-GMV	492.19 ± 21.50	477.69 ± 22.57	0.030^*^	479.35 ± 24.10	472.58 ± 16.82	0.130
N-WMV	341.53 ± 17.20	345.23 ± 18.99	0.562	345.10 ± 19.02	345.65 ± 19.75	0.540
TIV	1393.66 ± 146.02	1466.13 ± 159.41	0.105	1447.54 ± 151.24	1523.44 ± 176.88	0.227

* *P < 0.05*.

**Figure 2:**
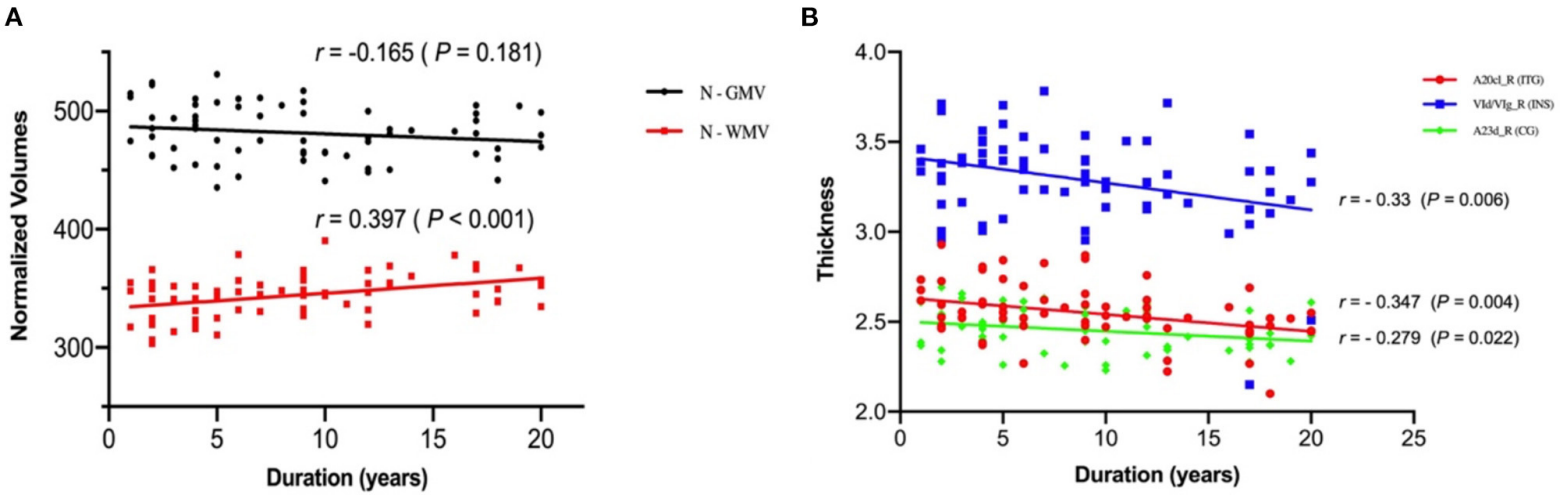
Correlation of the N-GMV, N-WMV and thickness with the duration of the juvenile myoclonic epilepsy patients. **(A)** The normalized volume of white matter is positively correlated with duration of disease. **(B)** The thicknesses of the right inferior temporal gyrus (ITG), insular gyrus (INS) and cingulate gyrus (CG) are negatively correlated with the duration of disease. N-GMV, normalized-GMV = (GMV/TIV) *1000; N-WMV, normalized-WMV = (WMV/TIV) *1000.

As shown in Figure 2, the N-WMV was positively correlated with duration of disease while the thicknesses of the right inferior temporal gyrus (ITG), insular gyrus (INS) and cingulate gyrus (CG) presented negative correlations with the duration.

**Figure 3:**
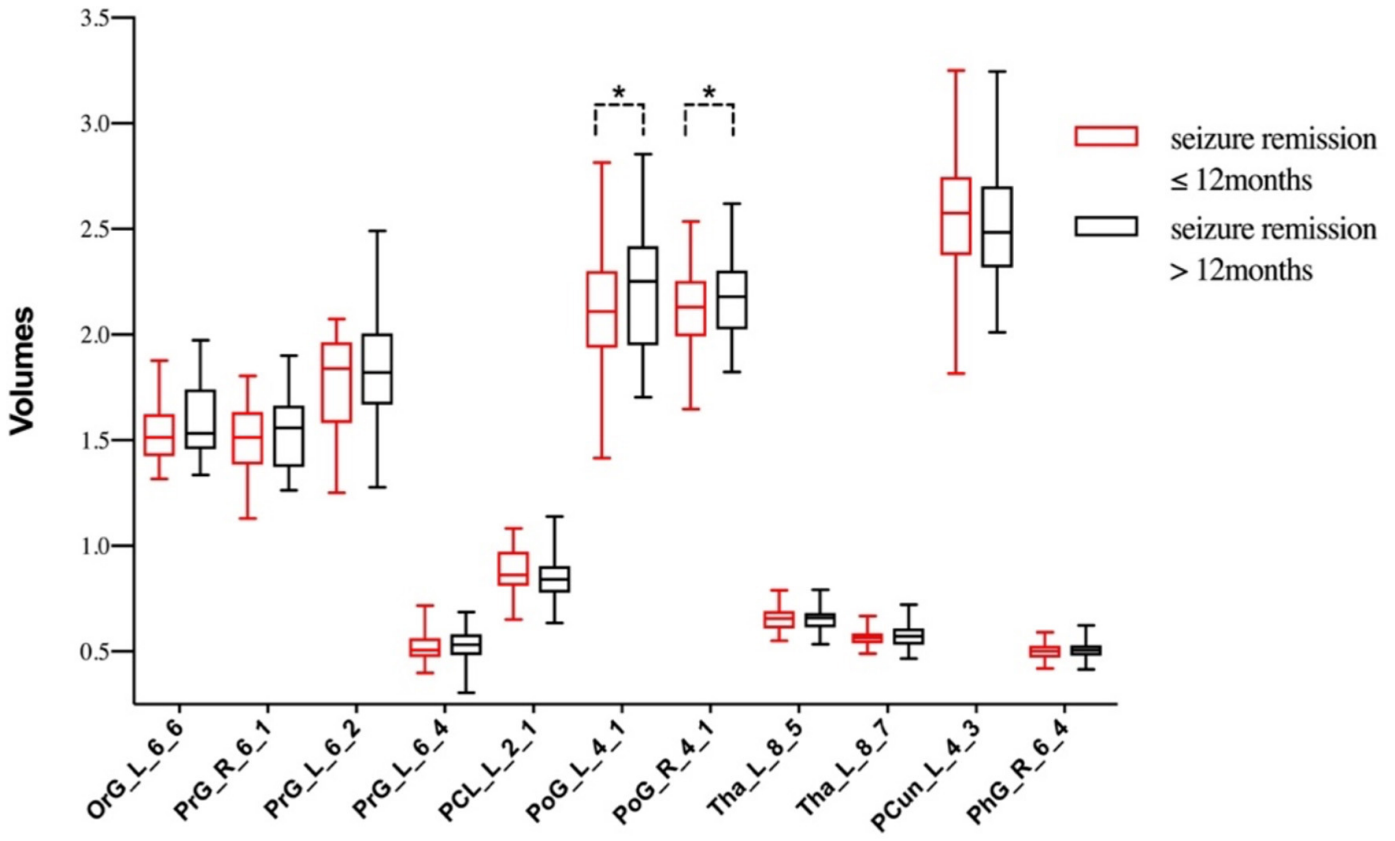
The differences of gray matter volumes between patients with different seizure remission time. *P < 0.05, TIV, age and duration as covariates.

From another perspective, Figure 3 reflected the relationship between seizure remission time and gray matter volumes. Of the 67 patients, 30 patients (44.8%) reported seizure remission for more than 12 months, whereas 37 patients (55.2%) still experienced MS and/or GTCS and /or AS during the previous 12 months. Meanwhile, compared with the group of seizure free, the left orbital gyrus, bilateral pre/post-central gyrus and left thalamus represented a trend of volume reduction in patients with persistent seizures.

### Neuropsychological Outcomes

#### The Original Neuropsychological Tests' Scores of Both JME Patients and Healthy Control Subjects

Compared with 56 normal controls, JME subjects got lower average scores in in Raven's Standard Progressive Matrices, Wisconsin Card Sorting Test, Visual Research Task, Visual Tracing Task, AVLT Immediate Memory, AVLT Delayed Memory, Digit Span, Digital n-back Test, Spatial n-back Test, Choice Reaction Time, Word Discrimination Test, Three-dimensional Mental Rotation, Complex Subtraction Test and Visual Perception Task. The differences were statistically significant (*P* < 0.05) (Table 5). The results indicated that impaired cognitive domains in JME patients include executive function, working memory, attention, psychomotor speed, visuospatial function, semantic knowledge, calculation, and visual perception abilities.

**Table 5:** Comparison of scores on neuropsychological test between two groups (Mean ± SD).

**Dimension**	**Neuropsychological test**	**JME (*N* = 67)**	**Controls (*N* = 56)**	***P*-Value**
Executive function	Wisconsin Card Sorting Test	75.89 ± 11.19	84.39 ± 5.38	< 0.001^*^
	Raven's Standard Progressive Matrices	21.91 ± 6.26	28.18 ± 5.55	< 0.001^*^
Attention	Visual Research Task	32.55 ± 38.11	67.43 ± 14.36	< 0.001^*^
	Visual Tracing Task	14.34 ± 5.69	16.95 ± 5.33	0.010^*^
Memory	AVLT Immediate Memory	37.11 ± 6.52	42.73 ± 4.61	< 0.001^*^
	AVLT Delayed Memory	12.72 ± 1.77	13.89 ± 1.29	< 0.001^*^
	AVLT Recognition Memory	13.13 ± 1.29	13.27 ± 2.03	0.442
	Digit Span	8.24 ± 1.84	9.4 ± 1.46	< 0.001^*^
	Digital n-back Test	68.19 ± 17.99	86.71 ± 12.58	< 0.001^*^
	Spatial n-back Test	22.89 ± 12.73	27.70 ± 11.40	0.029^*^
Psychomotor speed	Choice Reaction Time	396.33 ± 65.02	371.52 ± 63.73	0.035^*^
Visual perception	Visual Perception Task	72.97 ± 11.69	80.98 ± 7.85	< 0.001^*^
Visuospatial	Three-dimensional Mental Rotation	17.70 ± 15.30	24.03 ± 9.86	< 0.001^*^
Language	Word Discrimination Test	35.21 ± 8.69	40.39 ± 6.07	< 0.001^*^
Arithmetic calculation	Complex Subtraction Test	23.07 ± 5.99	27.55 ± 5.17	< 0.001^*^

* *Significantly different from control group (P < 0.05)*.

Considering the effects of drugs on cognition, the patients were divided into valproate group and non-valproate group according to their medication (Supplementary Table 5). Of the 67 patients, 37 (55.2%) were taking sodium valproate, and the remaining 30 patients were taking lamotrigine and/or levetiracetam. Differences in neuropsychological tests' scores between valproate and non-valproate groups were not statistically significant (*P* > 0.05).

#### Cognitive Impairments and Structural Abnormalities

Correlation analyses and linear regression analyses were performed on cognitive outcomes and the gray matter volume of each brain region, respectively. Then we summarized the Pearson correlation coefficient and *P*-value in Supplementary Tables 2, 3. It could be concluded from the tables that patients with reduced left or bilateral frontal lobe volumes have significantly worse executive function, attention, working memory, psychomotor speed and visuospatial function. Specifically, the executive function involved the dorsolateral frontal region, orbital gyrus, the lateral temporal region, and the left inferior parietal lobule; in addition, the volumes of insula, left cingulate gyrus, bilateral basal ganglia, and bilateral thalamus were also associated with executive function. Brain regions associated with attention included the entire frontal lobe, lateral temporal lobe, parietal lobe, the limbic system (insula and cingulate gyrus), and the left subcortical basal ganglia. The immediate and short-term memory were associated with the temporal lobe (left significantly), the left cingulate, basal ganglia, and thalamus. Spatial working memory was mainly related to frontal, temporal and parietal cortex, while the occipital cortex, limbic system and thalamus were also associated. The calculation ability was mainly associated with left frontal, temporal, parietal lobe, and bilateral subcortical regions. Moreover, the visuospatial ability and psychomotor speed were predominantly involved with the fronto-temporo-parietal cortex, and the limbic regions and subcortical thalamic regions. Thus, in addition to the frontoparietal lobe, other regions such as the temporal lobe, cingulate gyrus, subcortical basal ganglia, and thalamus may also be closely related to the cognitive impairments of JME.

## Discussion

Meencke and Janz first descripted the minimal malformations of cortical development in patients with JME, which reported as “microdysgenesis” (18). Since then, a number of studies have reported the cerebral structural changes in JME patients (19). These changes have mostly been localized to the frontal lobe and the thalami (14). More widespread cortical thinning involving the temporal, parietal, and occipital lobes was observed by surface-based morphometry (6, 20–22). Woermann found an increased mesiofrontal gray matter concentration in JME patients using voxel-based morphometry (VBM) (23, 24). Some researchers suggested that these findings of increased gray matter concentration could reflect microdysgenesis (6, 7). However, in our study, we came to the opposite conclusion that the gray matter concentration and volumes were mainly reduced, which was consistent with the decrease of cortical thickness. Compared with 56 normal controls, the GMC was significantly decreased in the bilateral pre- and post-central gyri, the right anterior cingulate gyrus, the left posterior orbital region, the bilateral hippocampus, the bilateral caudate nucleus, and the bilateral occipital region of the 67 JME patients, while we found no brain regions with increased GMC. The volumetry evaluation exhibited a significant GMV reductions in the frontal lobe (bilateral pre-central gyrus, left orbital gyrus and left paracentral lobule), parietal lobe (bilateral post-central gyrus), and subcortical regions (left basal ganglia and left amygdala). At the same time, JME patients had significant GMV increases in the left precuneus and right parahippocampal gyrus, which was not reported in previous studies. JME has been associated with an abnormality in frontal, motor regions and thalamocortical network (15, 25–27). The above results of structural analysis demonstrated that JME had not only definite evidence of structural abnormalities of the frontal lobe but also showed structural abnormalities of the thalamus, basal ganglia, and temporal-parietal-occipital lobe, suggesting that JME may have pathophysiological mechanisms other than thalamic-frontal circuit.

On the other hand, we found a positive correlation between N-WMV and the duration of disease while the thicknesses of right inferior temporal gyrus, insular gyrus and cingulate gyrus were negatively correlated with duration of disease, which may suggest the progressive damage of the brain in JME patients. An SPECT study on patients with JME reported that regional cerebral blood flow (rCBF) in frontal lobe was negatively correlated with the duration of the disease (28). Furthermore, studies reported that patients with JME showed increased local spontaneous activity in the paracentral lobe, and there was a significant positive correlation with the age of onset. These results may reflect the influence of epilepsy on the neuroplasticity of JME patients. Patients with epilepsy at an earlier onset age may have a better compensation mechanism for growth and development, and can adapt and regulate epileptic activities more easily than a mature brain (29). These findings suggest that frontal lobe dysfunction in JME may change over time.

In our study, 73.1% of the patients showed abnormal EEG findings, and the discharges were mainly concentrated in the frontal lobe. The results in Table 4 indicated that patients with normal EEG patterns exhibit significantly increased N-GMV compared to those with abnormal EEG discharges. It's generally believed that the characteristic generalized spike and wave discharges implicate thalamo–cortical interactions (30). In animal models of idiopathic generalized epilepsy, abnormal activity in both thalamic and cortical structures is needed for generalized spike and wave generation (31). There was increasing evidence indicating that the basal ganglia may play an essential role in the generalized spike waves or seizures of IGE (32, 33). The EEG-fMRI studies have shown that patients with IGE had reduced blood-oxygenation-dependent activities in basal ganglia, which was associated with generalized spike and slow waves (34–36). These conclusions also corroborated our findings of cortical abnormalities in the basal ganglia region.

The cognitive deficits in JME patients have been investigated in various studies. These studies found impairments in various cognitive domains, including executive functions, working memory, attention, phonemic fluency, semantic fluency, processing speed, and visuospatial perception (37). However, the results of many studies were inconsistent, among which the deficits of working memory, executive function, processing speed, and attention were more common in JME subjects. Our study reported that patients with JME had unambiguous dysfunctions across a range of cognitive domains which involved attention, executive function, short-term memory/working memory, psychomotor speed, semantic knowledge, calculation, visuospatial function and visual perception abilities. The impairments in calculation, visual perception and short-term memory of JME patients were rare in previous studies, while delayed memory was preserved. These results further confirmed the extensive cognitive impairments of JME beyond frontal lobe function. Medication was often considered as a potential factor affecting cognitive function in patients with epilepsy, mainly in attention, alertness, psychomotor speed, and memory. Research by Sommerbeck found that VPA caused mild cognitive impairment, which was more pronounced at high blood levels (38). Carbamazepine, lamotrigine, and levetiracetam had less influence on cognitive function (39). Considering that the other two drugs had little effect on cognition, the patients were divided into valproate group and non-valproate group according to their medication. However, we found no differences in cognitive outcomes between the two groups (Supplementary Table 5). There were great differences in cognitive results between different studies, which may depend on the selection of patients. Epileptic attacks, heredity and other factors may have a specific impact on cognitive function. In the future, our research will try to further include new-onset drug-naive JME patients to explore the abnormalities of cognitive function and brain structure.

Executive function and attention are the most consistent deficits in JME patients (40). Some authors have verified that JME subjects showed decreased connectivity between the prefrontal and fronto-polar regions, possibly accounting for dysfunction of cognitive frontal lobe and impairment in executive function (20). Caeyenberghs and co-investigators found a subnetwork of increased connectivity in people with JME (15). The nodes of the network comprised the primary motor cortex, precuneus, bilateral parietal/post-central gyrus, subcortical regions and right hippocampus. Meanwhile, there was a significant association between this subnetwork and auditory memory, verbal fluency, and executive function tasks (15). In general, the brain network abnormalities in these brain areas were obvious. Our findings based on structural imaging were also similar to this conclusion. In our study, the structural abnormalities of JME population were mainly located in the pre- and post-central gyrus, posterior orbital region, anterior cingulate gyrus, basal ganglia, thalamus and hippocampus. These regions were closely associated with impaired cognitive function in JME patients, as shown in Supplementary Table 3. Traditionally, the executive function and working memory mainly depend on the frontal lobe, and related reports have been repeatedly mentioned in other studies. The visual attention and visuospatial impairments were associated with abnormalities in the dorsal visual pathway (41). Studies have demonstrated that the dorsal visual pathway is associated with a variety of spatial processes, including spatial orientation, spatial attention, and mental rotation (41). The posterior parietal network plays an essential role in the repositioning of visual attention. Furthermore, imaging studies have shown that the prefrontal network is involved in various dorsal pathway functions; for example, spatial working memory and attention depend on network connectivity in the dorsolateral prefrontal cortex (DLPFC) and the posterior parietal cortex (42–44); neural networks associated with mental rotation include the right upper parietal and premotor areas (45, 46). The results of our correlation analysis between structure and cognitive function could also explain these theories to some extent.

Other structural imaging analysis confirmed that both gray matter volume and microstructural integrity of the posterior cingulate cortex were related to mental flexibility (14). In a diffusion MRI tractography analysis, connectivity between post-central gyrus and precuneus was positively associated with verbal IQ, and verbal memory scores (15). In addition to the role of the cerebral cortex in cognition, the subcortical areas such as the basal ganglia and thalamus cannot be ignored. Thalamus relays information from cerebellar interposed nucleus and dentate nuclei to cerebral cortex composing the cerebellum related thalamocortical (CTC) pathway which is mainly involved in motor function as well as cognitive and affective function (47). The ventral-anterior thalamic nucleus and the ventrolateral nucleus receives information from globus pallidus, the primary output nucleus of basal ganglia, forming the basal ganglia related thalamocortical (BTC) pathway. The BTC pathway plays a decisive role in motor initiation and learning (48). Besides, the cerebellum and basal ganglia have also been said to be associated with motor, cognitive and affective dysfunctions in IGE (49). Consistent with our study in JME, basal ganglia and thalamus were associated with most cognitive functions. Our work thus suggested morphometric and functional abnormalities in JME extending beyond the classically involved fronto-cortico-thalamic or fronto-parietal systems and supported functional relevance of structural alterations in temporal lobe and basal ganglia. Yet, more precise conclusions require further functional MRI studies to verify the relationship between structural and cognitive networks.

## Conclusions

Juvenile myoclonic epilepsy patients showed complex structural abnormalities in the frontal lobe, parietal lobe and subcortical areas. The study also concluded a synergistic change between structural and cognitive networks.

Nevertheless, we cannot exclude the variability of abnormalities in individual patients. In the future, we can improve the self-control cohort study to compare the brain volume changes of the same patient with the course of disease prolonging. Moreover, the role of genetic factors in structure and cognition may also be considered.

## Data Availability Statement

The datasets presented in this article are not readily available due to privacy restrictions. Request to access the datasets should be directed to the corresponding author.

## Ethics Statement

The studies involving human participants were reviewed and approved by Xuanwu Hospital Capital Medical University Ethics Committees. Written informed consent to participate in this study was provided by the participants' legal guardian/next of kin.

## Author Contributions

WS: study conception and design. JZ: acquisition, analysis and interpretation of data, and drafting paper. DW, HY, HL, YJ, and HL: acquisition of data. ZZ and JL: imaging acquisition and methodological guidance. All authors contributed to the article and approved the submitted version.

## Funding

This work was supported by Beijing Hospitals Authority Clinical Medicine Development of special funding support (grant number: XMLX202117), The National Key Research and Development Project of China (grant number: 2018YFC1315204) and National Natural Science Foundation of China (grant number: 81571267).

## Conflict of Interest

The authors declare that the research was conducted in the absence of any commercial or financial relationships that could be construed as a potential conflict of interest.

## Publisher's Note

All claims expressed in this article are solely those of the authors and do not necessarily represent those of their affiliated organizations, or those of the publisher, the editors and the reviewers. Any product that may be evaluated in this article, or claim that may be made by its manufacturer, is not guaranteed or endorsed by the publisher.
