# Supplementary material for: Correlations Between Structural Brain Abnormalities, Cognition and Electroclinical Characteristics in Patients With Juvenile Myoclonic Epilepsy
Source: Front Neurol. 2022 May 16;13:883078. doi: 10.3389/fneur.2022.883078 (PMC9149597; doi:10.3389/fneur.2022.883078)
Supplement: Supplementary file 1 [file Table_1.docx]

**Supplementary Table 1.**

The morphologic parameters (average thickness) in patients with JME relative to controls

| Surface parameters | Lobe | BNA Cortical area | MNI coordinates | JME (Mean ± SD) | Controls (Mean ± SD) | *P* - value^a^ |
| --- | --- | --- | --- | --- | --- | --- |
| **Thickness (mm)** | SFG | A8m_L/R | -5, 15, 54/ 7, 16, 54 | 2.90 ± 0.13/ 2.90 ± 0.15 | 2.96 ± 0.12/ 2.96 ± 0.12 | < 0.001/ < 0.001 |
|  |  | A8dl_L/R | -18, 24, 53/ 22, 26, 51 | 2.76 ± 0.13/ 2.72 ± 0.15 | 2.83 ± 0.11/ 2.78 ±0.13 | < 0.001/< 0.001 |
|  |  | A6dl_L/R | -18, -1, 65/ 20, 4, 64 | 2.73 ± 0.13/ 2.80 ± 0.15 | 2.82 ± 0.12/ 2.87 ± 0.13 | < 0.001/ 0.001 |
|  |  | A6m_L/R | -6, -5, 58/ 7, -4, 60 | 2.76 ± 0.15/ 2.77 ± 0.15 | 2.83 ± 0.12/ 2.84 ± 0.13 | < 0.001/< 0.001 |
|  |  | A9m_L/R | -5, 36, 38/ 6, 38, 35 | 2.88 ± 0.11/ 2.86 ± 0.10 | 2.93 ± 0.12/ 2.92 ± 0.10 | < 0.001/< 0.001 |
|  | MFG | A9/46d_L/R | -18, 24, 53/ 30, 37, 36 | 2.44 ± 1.24/ 2.50 ± 0.12 | 2.49 ± 0.12/2.56 ± 0.12 | 0.003/ 0.001 |
|  |  | IFJ_L/R | -42, 13, 36/ 42, 11, 39 | 2.55 ± 0.13/ 2.56 ± 0.14 | 2.61 ± 0.10/ 2.59 ± 0.11 | < 0.001/ 0.003 |
|  |  | A46_L/R | -28, 56, 12/ 28, 55, 17 | 2.31 ± 0.13/ 2.36 ± 0.13 | 2.36 ± 0.11/ 2.40 ± 0.10 | 0.005/ 0.015 |
|  |  | A9/46v_L/R | -41, 41, 16/ 42, 44, 14 | 2.38 ± 0.12/ 2.41 ± 0.13 | 2.44 ± 0.11/ 2.44 ± 0.11 | 0.001/ 0.006 |
|  |  | A8vl_L/R | -33, 23, 45/ 42, 27, 39 | 2.61 ± 0.15/ 2.57 ± 0.14 | 2.66 ± 0.11/ 2.62 ± 0.13 | < 0.001/ 0.002 |
|  |  | A6vl_L/R | -32, 4, 55/ 34, 8, 54 | 2.62 ± 0.13/ 2.65 ± 0.15 | 2.69 ± 0.12/ 2.73 ± 0.11 | < 0.001/ < 0.001 |
|  | IFG | A44d_L/R | -46, 13, 24/ 45, 16, 25 | 2.55 ± 0.14/ 2.48 ± 0.14 | 2.60 ± 0.11/ 2.52 ±0.13 | 0.001/ 0.001 |
|  |  | IFS_L/R | -47, 32, 14/ 48, 35, 13 | 2.40 ± 0.12/ 2.43 ± 0.12 | 2.50 ± 0.09/ 2.46 ± 0.11 | < 0.001/ 0.004 |
|  |  | A44v_L/R | -52, 13, 6/ 54, 14, 11 | 2.64 ± 0.12/ 2.61 ± 0.13 | 2.69 ± 0.11/ 2.65 ± 0.11 | 0.008/ 0.008 |
|  | OrG | A12/47o_L/R | -36, 33, -16/ 40, 39, -14 | 2.72 ± 0.13/ 2.74 ± 0.15 | 2.77 ± 0.12/ 2.78 ± 0.14 | 0.011/ 0.019 |
|  |  | A11l_L/R | -23, 38, -18/ 23, 36, -18 | 2.59 ± 0.12/ 2.64 ± 0.13 | 2.65 ± 0.13/ 2.70 ± 0.11 | 0.009/ 0.004 |
|  | PrG | A4hf_L/R | -49, -8, 39/ 55, -2, 33 | 2.47 ± 0.14/ 2.44 ± 0.14 | 2.54 ± 0.10/ 2.52 ± 0.12 | < 0.001/ < 0.001 |
|  |  | A6cdl_L/R | -32, -9, 58/ 33, -7, 57 | 2.62 ± 0.14/ 2.63 ± 0.13 | 2.69 ± 0.11/ 2.69 ± 0.11 | < 0.001/ < 0.001 |
|  |  | A4ul_L/R | -26, -25, 63/ 34, -19, 59 | 2.20 ± 0.17/ 2.29 ± 0.16 | 2.28 ± 0.14/ 2.37 ± 0.13 | < 0.001/ < 0.001 |
|  |  | A4t_L/R | -13, -20, 73/ 15, -22, 71 | 2.48 ± 0.22/ 2.36 ± 0.20 | 2.61 ± 0.17/ 2.44 ± 0.19 | < 0.001/ 0.004 |
|  |  | A4tl_L/R | -52, 0, 8/ 54, 4, 9 | 2.71 ± 0.13/ 2.74 ± 0.12 | 2.75 ± 0.12/ 2.77 ± 0.11 | 0.009/ 0.031 |
|  |  | A6cvl_L/R | -49, 5, 30/ 51, 7, 30 | 2.67 ± 0.13/ 2.65 ± 0.14 | 2.74 ± 0.11/ 2.69 ± 0.11 | < 0.001/ 0.001 |
|  | PCL | A1/2/3ll_L/R | -8, -38, 58/ 10, -34, 54 | 2.22 ± 0.12/ 2.33 ± 0.12 | 2.29 ± 0.10/ 2.38 ± 0.10 | < 0.001/ < 0.001 |
|  |  | A4ll_L/R | -4, -23, 61/ 5, -21, 61 | 2.56 ± 0.17/ 2.54 ± 0.16 | 2.68 ± 0.13/ 2.63 ± 0.17 | < 0.001/ < 0.001 |
|  | STG | A38m_R/ A38l_R | 31, 15, -34/ 47, 12, -20 | 3.33 ± 0.42/ 3.16 ± 0.25 | 3.56 ± 0.33/ 3.26 ± 0.18 | 0.004/ 0.008 |
|  | MTG | A21c_L/R | -65, -30, -12/ 65, -29, -13 | 2.77 ± 0.16/ 2.70 ± 0.15 | 2.79 ± 0.14/ 2.75 ± 0.13 | 0.039/ 0.006 |
|  |  | A21r_R | 51, 6, -32 | 3.06 ± 0.15 | 3.12 ± 0.12 | 0.020 |
|  | ITG | A20r_L | -43, -2, -41 | 2.93 ± 0.21 | 3.02 ± 0.16 | 0.019 |
|  |  | A20il_R/ A20cl_R | 55, -11, -32/ 61, -40, -17 | 2.81 ± 0.16/ 2.55 ± 0.16 | 2.85 ± 0.15/ 2.60 ± 0.15 | 0.027/ 0.011 |
|  | FuG | A20rv_R | 33, -15, -34 | 2.62 ± 0.28 | 2.73 ± 0.25 | 0.026 |
|  | PhG | A35/36r_R/ TL_R | 28, -8, -33/ 30, -30, -18 | 3.12 ± 0.84/ 2.27 ± 0.49 | 3.40 ± 0.59/ 2.45 ± 0.34 | 0.037/ 0.016 |
|  | SPL | A7r_L/R | -16, -60, 63/ 19, -57, 65 | 2.28 ± 0.13/ 2.26 ± 0.12 | 2.31 ± 0.14/ 2.31 ± 0.15 | 0.012/ 0.003 |
|  |  | A7c_L/R | -15, -71, 52/ 19, -69, 54 | 2.19 ± 0.12/ 2.21 ± 0.12 | 2.22 ± 0.12/ 2.25 ± 0.10 | 0.030/ 0.013 |
|  |  | A5l_L/R | -33, -47, 50/ 35, -42, 54 | 2.20 ± 0.12/ 2.17 ± 0.13 | 2.23 ± 0.09/ 2.20 ± 0.12 | 0.005/ 0.025 |
|  |  | A7pc_L/R | -22, -47, 65/ 23, -43, 67 | 2.17 ± 0.11/ 2.17 ± 0.11 | 2.22 ± 0.12/ 2.20 ± 0.12 | 0.001/ 0.024 |
|  | IPL | A39rd_L/R | -38, -61, 46/ 39, -65, 44 | 2.38 ± 0.12/ 2.38 ± 0.11 | 2.40 ± 0.10/ 2.41 ± 0.11 | 0.035/ 0.005 |
|  |  | A40rd_L/R | -51, -33, 42/ 47, -35, 45 | 2.42 ± 0.12/ 2.31 ± 0.11 | 2.44 ± 0.10/ 2.34 ± 0.10 | 0.01/ 0.004 |
|  |  | A39rv_R/ A40rv_R | 53, -54, 25/ 55, -26, 26 | 2.49 ± 0.10/ 2.55 ± 0.11 | 2.52 ± 0.11/ 2.58 ± 0.11 | 0.003/ 0.005 |
|  | Pcun | A7m_L/R | -5, -63, 51/ 6, -65, 51 | 2.29 ± 0.14/ 2.32 ± 0.13 | 2.34 ± 0.12/ 2.37 ± 0.13 | 0.007/ 0.001 |
|  |  | A5m_L/R | -8, -47, 57/ 7, -47, 58 | 2.29 ± 0.12/ 2.32 ± 0.10 | 2.34 ± 0.10 /2.37 ± 0.10 | 0.002/ < 0.001 |
|  |  | A31_L/R | -6, -55, 34/ 6, -54, 35 | 2.43± 0.12/ 2.39 ± 0.12 | 2.46 ± 0.10/ 2.45 ± 0.10 | 0.018/ < 0.001 |
|  | PoG | A1/2/3ulhf_L/R | -50, -16, 43/ 50, -14, 44 | 2.00 ± 0.12/ 1.97 ± 0.12 | 2.06 ± 0.12/ 2.05 ± 0.11 | < 0.001/ < 0.001 |
|  |  | A2_L/R | -46, -30, 50/ 48, -24, 48 | 2.19 ± 0.13/ 2.19 ± 0.11 | 2.24 ± 0.12/ 2.24 ± 0.12 | 0.004/ 0.003 |
|  | INS | vla_R/ vId/vIg_R | 33, 14, -13/ 39, -2, -9 | 3.11± 0.40/ 3.29 ± 0.26 | 3.30 ± 0.18/ 3.38 ± 0.20 | < 0.001/ 0.009 |
|  | CG | A23d_R/ A32p_R | 4, -37, 32/ 5, 28, 27 | 2.45 ± 0.11/ 2.72 ± 0.15 | 2.49 ± 0.10/ 2.80 ± 0.14 | 0.018/ 0.002 |
|  |  | A24cd_R | 4, 6, 38 | 2.65 ± 0.15 | 2.70 ± 0.16 | 0.034 |
|  | MVOcC | rCunG_L/R | -5, -81, 10/ 7, -76, 11 | 1.81 ± 0.12/ 1.81 ± 0.11 | 1.84 ± 0.09/ 1.85 ± 0.10 | 0.019/ 0.002 |
|  |  | cCunG_L/R | -6, -94, 1/ 8, -90, 12 | 1.77 ± 0.12/ 1.79 ± 0.10 | 1.82 ± 0.10/ 1.85 ± 0.11 | 0.007/ 0.002 |
|  |  | rLinG_R/ vmPOS_R | 18, -60, -7/ 15, -63, 12 | 2.25 ± 0.13/ 2.02 ± 0.11 | 2.28 ± 0.12/ 2.04 ± 0.10 | 0.019/ 0.035 |
|  | LOcC | mOccG_R/ msOccG_L | 34, -86, 11/ -11, -88, 31 | 2.14 ± 0.11/ 2.00 ± 0.12 | 2.19 ± 0.10/ 2.04 ± 0.11 | 0.001/ 0.020 |

L, left; R, right; SFG, Superior frontal gyrus; MFG, Middle frontal gyrus; IFG, Inferior frontal gyrus; OrG, Orbital gyrus; PrG, Precentral gyrus; PCL, Paracentral lobule; STG, Superior temporal gyrus; MTG, Middle temporal gyrus; ITG, Inferior temporal gyrus; FuG, Fusiform Gyrus; PhG, Parahippocampal Gyrus; SPL, Superior parietal lobule; IPL,Inferior parietal Lobule; Pcun, Precuneus; PoG, Postcentral gyrus; INS, Insular gyrus; CG, Cingulate gyrus; MVOcC, MedioVentral Occipital Cortex; LOcC, lateral Occipital Cortex. ^a^ Covariance analysis: TIV (total intracranial volume), age and gender as covariates.
