# Supplementary material for: Correlations Between Structural Brain Abnormalities, Cognition and Electroclinical Characteristics in Patients With Juvenile Myoclonic Epilepsy
Source: Front Neurol. 2022 May 16;13:883078. doi: 10.3389/fneur.2022.883078 (PMC9149597; doi:10.3389/fneur.2022.883078)
Supplement: Supplementary file 2 [file Table_2.docx]

**Supplementary Table 2.**

Correlation of neuropsychological tests’ scores with the volumes of brain regions with statistical differences in gray matter in JME.

| Neuropsychological test | OrG_L_6_6 | PrG_L_6_2 | PrG_L_6_4 | PCL_L_2_1 | PrG_R_6_1 | PoG_L_4_1 | PoG_R_4_1 | Tha_L_8_5 | Tha_L_8_7 | PCun_L_4_3 | PhG_R_6_4 |
| --- | --- | --- | --- | --- | --- | --- | --- | --- | --- | --- | --- |
|  | Pearson Correlation/ *P*-value | | | | | | | | | | |
| Raven's Standard Progressive Matrices | 0.231/0.059 | 0.235/0.055 | 0.147/0.235 | -0.08/0.522 | **.281^*^/0.021^#^** | 0.122/0.324 | 0.182/0.14 | **.439^**^/<0.001^#^** | **.420^**^/<0.001^#^** | 0.098/0.431 | **.246^**^/0.045^#^** |
| Visual Research Task | 0.102/0.410 | 0.117/0.346 | 0.183/0.138 | 0.165/0.182 | 0.073/0.555 | 0.159/0.199 | 0.009/0.944 | 0.16/0.195 | 0.18/0.145 | 0.222/0.07 | 0.076/0.54 |
| Visual Tracing Task | **.298^*^/0.014^#^** | 0.196/0.111 | **.341^**^/0.005^#^** | 0.237/0.054 | 0.229/0.062 | **.301^*^/0.013^#^** | 0.142/0.253 | 0.153/0.215 | 0.111/0.373 | 0.172/0.163 | 0.17/0.169 |
| AVLT Immediate Memory | 0.158/0.202 | 0.179/0.147 | 0.135/0.278 | 0.084/0.498 | **.347^**^/0.004^#^** | **.279^*^/0.022^#^** | **.270^*^/0.027^#^** | **.308^*^/0.011^#^** | 0.188/0.127 | 0.135/0.276 | 0.065/0.604 |
| AVLT Delayed Memory | 0.136/0.272 | 0.169/0.171 | 0.098/0.432 | 0.129/0.298 | 0.225/0.067 | **.251^*^/0.04^#^** | 0.173/0.161 | **.288^*^/0.018^#^** | 0.165/0.181 | 0.228/0.064 | 0.081/0.514 |
| Digit Span | 0.086/0.491 | 0.091/0.464 | 0.178/0.151 | 0.118/0.342 | 0.221/0.072 | 0.166/0.179 | **.305^*^/0.012^#^** | 0.222/0.071 | 0.173/0.161 | 0.104/0.402 | 0.115/0.356 |
| Digital n-back Test | 0.056/0.654 | 0.074/0.553 | 0.190/0.124 | 0.071/0.568 | 0.199/0.106 | 0.115/0.354 | 0.1/0.422 | **.311^*^/0.010^#^** | **.246^*^/0.045^#^** | -0.013/0.915 | 0.102/0.412 |
| Spatial n-back Test | **.403^**^/0.001^#^** | 0.188/0.127 | 0.223/0.070 | 0.139/0.260 | **.332^**^/0.006^#^** | **.383^**^/0.001^#^** | **.287^*^/0.018^#^** | **.293^*^/0.016^#^** | **.256^*^/0.037^#^** | **.327^**^/0.007^#^** | 0.231/0.06 |
| Choice Reaction Time | **-.426^**^/<.001^#^** | **-.378^**^/0.002^#^** | -0.128/0.304 | -0.135/0.276 | **-.271^*^/0.027^#^** | **-.295^*^/0.015^#^** | **-.248^*^/0.043^#^** | **-.358^**^/0.003^#^** | **-.367^**^/0.002^#^** | -0.15/0.227 | **-.289^*^/0.018^#^** |
| Visual Perception Task | 0.198/0.109 | 0.145/0.242 | 0.163/0.186 | 0.039/0.755 | 0.198/0.109 | 0.058/0.643 | 0.113/0.363 | 0.237/0.053 | 0.112/0.369 | 0.119/0.337 | 0.109/0.379 |
| Three-dimensional Mental Rotation | **.361^**^/0.003^#^** | **.318^*^/0.009^#^** | **.304^*^/0.012^#^** | **.291^*^/0.017^#^** | **.321^**^/0.008^#^** | **.332^**^/0.006^#^** | **.316^**^/0.009^#^** | **.426^**^/<0.001^#^** | **.385^**^/0.001^#^** | **.467^**^/<0.001^#^** | **.368^**^/0.002^#^** |
| Word Discrimination Test | 0.238/0.052 | **.283^*^/0.02^#^** | 0.066/0.597 | 0.16/0.196 | 0.204/0.098 | 0.171/0.167 | 0.097/0.434 | **.318^**^/0.009^#^** | **.284^*^/0.02^#^** | 0.232/0.059 | 0.21/0.089 |
| Complex Subtraction Test | 0.114/0.360 | 0.156/0.209 | 0.003/0.982 | 0.214/0.082 | **.289^*^/0.018^#^** | 0.15/0.226 | 0.147/0.234 | **.366^**^/0.002^#^** | **.330^**^/0.006^#^** | 0.203/0.099 | 0.165/0.181 |

^*/**^, Pearson Correlation; ^#^, *P* < 0.05
