# Supplementary material for: Correlations Between Structural Brain Abnormalities, Cognition and Electroclinical Characteristics in Patients With Juvenile Myoclonic Epilepsy
Source: Front Neurol. 2022 May 16;13:883078. doi: 10.3389/fneur.2022.883078 (PMC9149597; doi:10.3389/fneur.2022.883078)
Supplement: Supplementary file 3 [file Table_3.docx]

**Supplementary Table 3.**

Correlation of neuropsychological tests’ scores with the volumes of all brain subregions in JME.

| Neuropsychological test | Frontal lobe | | | | | | Temporal lobe | | | | | Parietal lobe | | | |
| --- | --- | --- | --- | --- | --- | --- | --- | --- | --- | --- | --- | --- | --- | --- | --- |
|  | SFG | MFG | IFG | OrG | PrG | PCL | STG | MTG | ITG | FuG | PhG | SPL | IPL | PCun | PoG |
| Raven's Standard Progressive Matrices |  | B^**,##^ | L^**,##^ | B^**,##^ |  |  | B^**,##^ | B^**,##^ | L^**,##^ | L^**,##^ |  |  | L^**,##^ |  |  |
| Visual Research Task |  |  |  |  | L^*,#^ |  | L^*,#^ |  | L^*,#^ |  |  | L^*,#^ | L^*,#^ |  |  |
| Visual Tracing Task | B^**,##^ | B^**,##^ | B^**,##^ | B^**,##^ | B^**,##^ | L^**,##^ | B^**,##^ | B^**,##^ | B^**,##^ |  |  | B^**,##^ | B^**,##^ | R^**,##^ | B^**,##^ |
| AVLT Immediate Memory |  |  |  |  |  |  |  | L^**,##^ | L^**,##^ | L^**,##^ |  |  |  |  |  |
| AVLT Delayed Memory |  |  |  |  |  |  |  | B^**,##^ | L^**,##^ |  |  |  |  |  |  |
| Digit Span |  |  |  |  |  |  |  |  | L^**,##^ |  |  | L^**,##^ |  |  |  |
| Digital n-back Test |  |  |  |  |  |  |  |  | L^**,##^ |  |  |  |  |  |  |
| Spatial n-back Test |  | B^**,##^ | L^**,##^ | B^**,##^ | B^**,##^ |  | B^**,##^ | B^**,##^ | B^**,##^ | B^**,##^ | B^**,##^ |  | B^**,##^ | L^**,##^ | L^**,##^ |
| Choice Reaction Time |  | B^**,##^ | B^**,##^ | B^**,##^ | L^**,##^ |  | B^**,##^ | B^**,##^ | B^**,##^ | L^**,##^ | B^**,##^ |  | B^**,##^ |  |  |
| Visual Perception Task | L^**,##^ |  |  |  | R^**,##^ |  |  | R^*,#^ | R^*,#^ |  |  | R^**,##^ |  |  |  |
| Three-dimensional Mental Rotation | B^**,##^ | B^**,##^ | B^**,##^ | B^**,##^ | B^**,##^ |  | B^**,##^ | B^**,##^ | B^**,##^ | B^**,##^ | B^**,##^ |  | B^**,##^ | B^**,##^ | B^**,##^ |
| Word Discrimination Test | L^**,##^ | R^**,##^ |  |  | L^**,##^ |  | R^**,##^ | R^**,##^ |  |  |  | L^**,##^ |  |  | B^**,##^ |
| Complex Subtraction Test | L^**,##^/ |  | L^**,##^ |  |  | L^*,#^ | L^**,##^ |  | L^**,##^ |  |  |  |  |  | L^**,##^ |

| Neuropsychological test | Occipital lobe | | Insular lobe | Cingulate gyrus | Subcortical area | | | |
| --- | --- | --- | --- | --- | --- | --- | --- | --- |
|  | MVOcC | LOcC |  |  | Amyg | Hipp | BG | Tha |
| Raven's Standard Progressive Matrices |  |  | B^**,##^ | L^**,##^ |  |  | B^**,##^ | B^**,##^ |
| Visual Research Task |  |  |  |  |  | B^**,##^ | L^*,#^ | L^**,##^ |
| Visual Tracing Task | L^**,##^ |  | B^**,##^ | B^**,##^ |  |  | B^**,##^ |  |
| AVLT Immediate Memory | L^**,##^ |  |  | L^**,##^ |  |  | L^**,##^ | L^**,##^ |
| AVLT Delayed Memory |  |  |  | L^**,##^ |  |  | L^**,##^ | L^**,##^ |
| Digit Span |  |  |  | R^**,##^ |  |  |  | R^**,##^ |
| Digital n-back Test |  |  |  |  |  |  | B^**,##^ |  |
| Spatial n-back Test | B^**,##^ | B^**,##^ | B^**,##^ | B^**,##^ | B^**,##^ | L^**,##^ |  | B^**,##^ |
| Choice Reaction Time | B^**,##^ |  | B^**,##^ | L^**,##^ | B^**,##^ | L^**,##^ | B^**,##^ | B^**,##^ |
| Visual Perception Task |  |  |  |  |  |  | R^**,##^ |  |
| Three-dimensional Mental Rotation | B^**,##^ |  | B^**,##^ |  | B^**,##^ |  |  | B^**,##^ |
| Word Discrimination Test |  |  | L^**,##^ | R^**,##^ |  |  | L^**,##^ | B^**,##^ |
| Complex Subtraction Test |  | R^**,##^ |  |  | B^**,##^ |  | B^**,##^ | B^**,##^ |

B, Bilateral; L, left; R, right. ^*/**^, Pearson Correlation; ^#^, *P* < 0.05; ^##^, *P* < 0.01.
