# Supplementary material for: Correlations Between Structural Brain Abnormalities, Cognition and Electroclinical Characteristics in Patients With Juvenile Myoclonic Epilepsy
Source: Front Neurol. 2022 May 16;13:883078. doi: 10.3389/fneur.2022.883078 (PMC9149597; doi:10.3389/fneur.2022.883078)
Supplement: Supplementary file 4 [file Table_4.docx]

**SupplementaryTable 4.**

Gray matter differences between patients with monotherapy and with polytherapy

|  |  | Volumes (Mean ± SD) (cm^3^) | |  |
| --- | --- | --- | --- | --- |
| Lobe | BNA label | Monotherapy (N = 44) | Polytherapy (N = 23) | *P* - value |
|  | GMV | 703.84 ± 65.38 | 678.31 ± 68.79 | 0.150 |
|  | WMV | 500.89 ± 56.63 | 492.12 ± 67.82 | 0.576 |
| Frontal lobe | OrG_L_6_6 | 2.28 ± 0.31 | 2.18 ± 0.30 | 0.194 |
|  | PrG_L_6_2 | 2.58 ± 0.41 | 2.58 ± 0.46 | 0.990 |
|  | PrG_L_6_4 | 2.11 ± 0.19 | 2.08 ± 0.23 | 0.960 |
|  | PCL_L_2_1 | 1.25 ± 0.14 | 1.23 ± 0.20 | 0.667 |
|  | PrG_R_6_1 | 2.24 ± 0.25 | 2.12 ± 0.29 | 0.079 |
| Parietal lobe | PoG_L_4_1 | 3.21 ± 0.42 | 2.90 ± 0.41 | 0.056 |
|  | PoG_R_4_1 | 3.11 ±0.31 | 3.01 ± 0.34 | 0.230 |
| Subcortical area | Amyg_L_2_2 | 0.45 ± 0.04 | 0.44 ± 0.05 | 0.764 |
|  | BG_L_6_1 | 2.00 ± 0.24 | 1.99 ± 0.25 | 0.398 |
|  | Tha_L_8_5 | 0.955 ± 0.095 | 0.927 ± 0.104 | 0.266 |
|  | Tha_L_8_7 | 0.825 ± 0.095 | 0.820 ± 0.107 | 0.846 |

Covariance analysis: TIV (total intracranial volume), age and gender as covariates. The volumes of patients with monotherapy were greater than those with polytherapy, but the differences were not statistically significant (*P*> 0.05).
