# Supplementary material for: Correlations Between Structural Brain Abnormalities, Cognition and Electroclinical Characteristics in Patients With Juvenile Myoclonic Epilepsy
Source: Front Neurol. 2022 May 16;13:883078. doi: 10.3389/fneur.2022.883078 (PMC9149597; doi:10.3389/fneur.2022.883078)
Supplement: Supplementary file 5 [file Table_5.docx]

**SupplementaryTable 5.**

Comparison of scores on Neuropsychological test between valproate and non-valproate groups (Mean ± SD)

| Dimension | Neuropsychological test | valproate (N = 37) | non-valproate (N = 30) | *P* - value |
| --- | --- | --- | --- | --- |
| Executive function | Wisconsin Card Sorting Test | 74.22 ± 11.01 | 77.97 ± 11.23 | 0.242 |
|  | Raven's Standard Progressive Matrices | 22.89 ± 5.00 | 20.70 ± 7.45 | 0.128 |
| Attention | Visual Research Task | 28.30 ± 38.50 | 37.80 ± 37.60 | 0.379 |
|  | Visual Tracing Task | 14.54 ± 5.64 | 14.10 ± 5.84 | 0.552 |
| Memory | AVLT Immediate Memory | 36.61 ± 6.61 | 37.75 ± 6.46 | 0.533 |
|  | AVLT Delayed Memory | 12.54 ± 1.82 | 12.93 ± 1.70 | 0.401 |
|  | AVLT Recognition Memory | 13.22 ± 1.27 | 13.03 ± 1.33 | 0.442 |
|  | Digit Span | 8.26 ± 1.83 | 8.22 ± 1.88 | 0.948 |
|  | Digital n-back Test | 68.27 ± 18.04 | 68.10 ± 18.25 | 0.933 |
|  | Spatial n-back Test | 21.57 ± 12.37 | 24.53 ± 13.18 | 0.403 |
| Psychomotor speed | Choice Reaction Time | 389.30 ± 57.19 | 408.33 ± 72.46 | 0.227 |
| Visual perception | Visual Perception Task | 72.03 ± 11.66 | 74.13 ± 11.83 | 0.457 |
| Visuospatial | Three-dimensional Mental Rotation | 18.24 ± 13.73 | 17.03 ± 17.26 | 0.947 |
| Language | Word Discrimination Test | 36.84 ± 6.91 | 33.20 ± 10.25 | 0.059 |
| Arithmetic calculation | Complex Subtraction Test | 22.73 ± 5.90 | 23.50 ± 6.17 | 0.881 |

Differences in neuropsychological tests’ scores between valproate and non-valproate groups were not statistically significant (*P*> 0.05).
